# Discovery and validation of methylation signatures in blood-based circulating tumor cell-free DNA in early detection of colorectal carcinoma: a case–control study

**DOI:** 10.1186/s13148-020-00985-4

**Published:** 2021-02-03

**Authors:** Jinke Sui, Xianrui Wu, Chenyang Wang, Guoqiang Wang, Chengcheng Li, Jing Zhao, Yuzi Zhang, Jianxing Xiang, Yu Xu, Weiqi Nian, Fuao Cao, Guanyu Yu, Zheng Lou, Liqiang Hao, Lianjie Liu, Bingsi Li, Zhihong Zhang, Shangli Cai, Hao Liu, Ping Lan, Wei Zhang

**Affiliations:** 1 Colorectal Surgery Department, Changhai Hospital, Naval Medical University, Changhai Road No.168, Yangpu District, Shanghai, 200433 China; 2 Colorectal Surgery Department, The Sixth Affiliated Hospital of Sun Yat-Sen University, Yuancun Erheng Road No. 26, Guangzhou, 510655 China; 3 Burning Rock Biotech, Guangzhou, China; 4 Phase I Ward, Chongqing University Cancer Hospital, Chongqing, China

**Keywords:** Colorectal carcinoma, Methylation, ctDNA, Early detection

## Abstract

**Background:**

Early detection of colorectal carcinoma (CRC) would help to identify tumors when curative treatments are available and beneficial. However, current screening methods for CRC, e.g., colonoscopy, may affect patients’ compliance due to the uncomfortable, invasive and time-consuming process. In recent decades, methylation profiles of blood-based circulating tumor DNA (ctDNA) have shown promising results in the early detection of multiple tumors. Here we conducted a study to investigate the performance of ctDNA methylation markers in early detection of CRC.

**Results:**

In total, 742 participants were enrolled in the study including CRC (*n* = 332), healthy control (*n* = 333), benign colorectal disease (*n* = 65) and advanced adenoma (*n* = 12). After age-matched and randomization, 298 participants (149 cancer and 149 healthy control) were included in training set and 141 (67 cancer and 74 healthy control) were in test set. In the training set, the specificity was 89.3% (83.2–93.7%) and the sensitivity was 88.6% (82.4–93.2%). In terms of different stages, the sensitivities were 79.4% (62.1–91.2%) in patients with stage I, 88.9% (77.3–95.8%) in patients with stage II, 91.4% (76.9–98.2%) in patients with stage III and 96.2% (80.3–99.9%) in patients with stage IV. Similar results were validated in the test set with the specificity of 91.9% (83.1–97.0%) and sensitivity of 83.6% (72.5–91.6%). Sensitivities for stage I-III were 87.0% (79.7–92.4%) in the training set and 82.5% (70.2–91.3%) in the test set, respectively. In the unmatched total population, the positive ratios were 7.8% (5.2–11.2%) in healthy control, 30.8% (19.9–43.5%) in benign colorectal disease and 58.3% (27.5–84.7%) in advanced adenoma, while the sensitivities of stage I–IV were similar with training and test sets. Compared with methylated SEPT9 model, the present model had higher sensitivity (87.0% [81.8–91.2%] versus 41.2% [34.6–48.1%], *P* < 0.001) under comparable specificity (90.1% [85.4–93.7%] versus 90.6% [86.0–94.1%]).

**Conclusions:**

Together our findings showed that ctDNA methylation markers were promising in the early detection of CRC. Further validation of this model is warranted in prospective studies.

## Background

Colorectal cancer (CRC) ranks third in terms of incidence and second in terms of mortality worldwide with an estimated 1.8 million new cases and 0.86 million deaths in 2018 [1]. Most CRC patients are advanced when diagnosed and the 5-year survival rate for advanced patients is only 12%, while for patients with localized disease or regional metastasis, the 5-year survival were 90% and 70%, suggesting a better prognosis for patients diagnosed earlier [2]. Thus, it is of great significance to early detect CRC when curative treatment is available. Moreover, the early detection of colorectal precancerous lesions before the development of invasive malignancy will help to decrease the risk of CRC [3, 4].

It is recommended by the American Cancer Society for people with average risk to perform colonoscopy, fecal immunochemical testing (FIT), fecal occult blood testing (FOBT), multi-target stool DNA test, flexible sigmoidoscopy or CT colonoscopy [5]. Though fecal-based tests are cheaper, less invasive than colonoscopy, the performance of fecal-based tests may be compromised by the presence of non-bleeding neoplasms and bleeding non-neoplastic conditions [6]. A multi-target stool DNA test outperformed FIT in sensitivity for detecting advanced precancerous lesions but had more false positive results [7]. So far, colonoscopy is the most accurate and effective approach for CRC screening [8]; however, the invasive, uncomfortable, irritating and time-consuming procedures of colonoscopy negatively affect patients’ compliance with recommended screening. Moreover, a large number of adenomas and serrated polyps may be missed due to the inadequate bowel preparation, the improper auxiliary techniques, and the size and morphology of adenomas [9], indicating that an accurate, noninvasive diagnostic test for both CRC and advanced precancerous lesions is highly desirable.

DNA methylation, characterized as the addition of a methyl group (CH_3_) at the C5 position of the cytosine ring by DNA methyltransferases (DNMTs), yielding 5-methylcytosine, which are heritable alterations in gene expression, involved in the differentiation, development, aging and pathogenesis of multiple cancers, including CRC [10, 11]. The epigenetic aberrations of circulating tumor DNA (ctDNA) can be detected in peripheral blood [12] and showed promising performance in clinical practice, including diagnosis [13–16], prognosis [13, 17] and drug resistance [17, 18]. Utilization of ctDNA-based DNA methylation to early detection may be a promising approach for CRC for several reasons. Firstly, cancer-specific methylation occurs early in tumorigenesis, appears to be stable, yields an amplified signal and can be assayed with high accuracy [19]. Secondly, current methods for the detection of circulating tumor DNA involve sequencing somatic mutations using ctDNA [20]; however, the major obstacles impeded the utility of mutations using ctDNA including the limited number of mutations available to distinguish tumor and normal in a cost-effective way and technical errors introduced during sequencing [21]. By contrast, hundreds to thousands of genes are thought to be aberrantly methylated in the average CRC epigenome; thus, DNA methylation profiling may have higher clinical sensitivity and dynamic range, multiple detectable methylation target regions and multiple altered CpG sites within each targeted genomic region [22]. Thirdly, the above characteristics of DNA methylation made it possible to detect DNA methylation via circulating tumor DNA in blood. Altogether, ctDNA-based DNA methylation may be an ideal noninvasive approach for early detection of CRC.

Several studies have been carried out attempting to evaluate the utility of ctDNA-based DNA methylation in the early detection of CRC. A recent study has demonstrated that ctDNA methylation had a sensitivity of 67.3% and a specificity of 99.3% in pre-specified set of 12 cancer types with stage I-III including CRC [23]. Another large, prospective trial has assessed the accuracy of circulating methylated SEPT9 for detecting CRC in 7941 patients using a commercially available assay yielding unsatisfied performance with low sensitivity and specificity of 48.2% and 91.5%, respectively [24]. A recent study further explored other methylation profiles and showed that ctDNA methylation profiles not only enabled early diagnosis but also prognosis prediction and screening for CRC [17]. In addition, Chen et al. demonstrated a blood-based cancer-specific methylation signature might help early detection of multiple cancer types up to four years prior to conventional diagnosis [25]. Altogether these studies demonstrated the promising utility of ctDNA methylation profiles in the early detection of CRC. Different panels have been reported attempting to improve the early detection of CRC in clinical practice; however, no definite biomarkers have been established and the appropriate panel for CRC early detection needs to be further studied.

Herein, we aimed to study the potential utility of ctDNA methylation in CRC early detection and to build an early detection model based on ctDNA methylation.

## Results

### Characteristics of participants

**Fig. 1:**
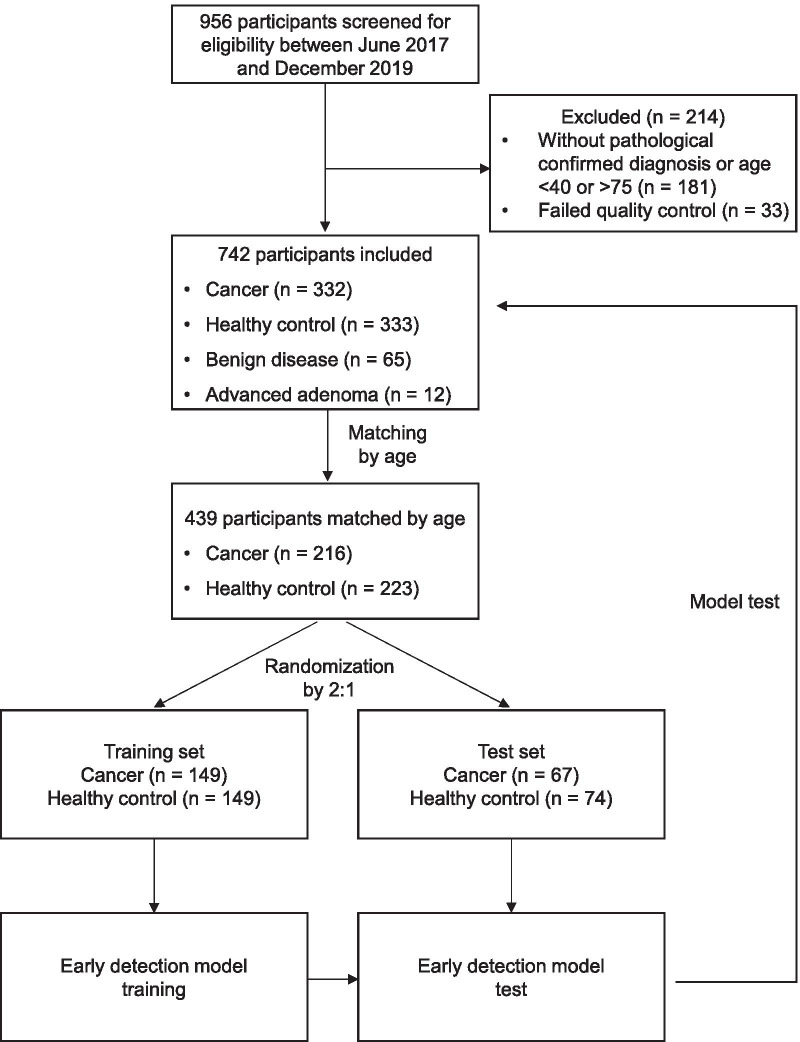
Study flow

In total, 742 participants were enrolled in the study including CRC (*n* = 332), healthy control (*n* = 333), benign colorectal disease (*n* = 65) and advanced adenoma (*n* = 12) (Fig. 1). After matched by age, 216 CRC patients and 223 healthy controls were then randomized into training set and test set with a 2:1 ratio (Fig. 1). In detail, 298 (149 cancer and 149 healthy control) participants were included in the training set and 141 (67 cancer and 74 healthy control) participants were in the test set.

The detailed characteristics of participants in training and test cohorts are demonstrated in Table 1. In the training set, there were 149 participants with CRC including 34 (23%) with stage I, 54 (36%) with stage II, 35 (23%) with stage III and 26 (17%) with stage IV. In test set, there were 67 participants with CRC including 17 (25%) with stage I, 25 (37%) with stage II, 15 (22%) with stage III and 10 (15%) with stage IV. Sex were relatively balanced in healthy controls (men: 54% in training set and 55% in test set), while the majority of CRC patients were men (67% men in training set and 64% men in test set), which was consistent with the epidemiology of CRC that incidence rate of CRC is higher in men than women [1]. Training and test sets were generally comparable with respect to age in the cancer and healthy controls groups (*P* > 0.05).

**Table 1:** Baseline characteristics for training and test sets

	Training set			Test set		
	Colorectal carcinoma (*n* = 149)	Healthy control (*n* = 149)	*P*	Colorectal carcinoma (*n* = 67)	Healthy control (*n* = 74)	*P*
Age, years						
Mean ± SD	57.5 ± 9.8	56.5 ± 5.6	0.26	58.1 ± 10.5	56.2 ± 4.9	0.18
Sex, *n* (%)			0.02			0.30
Male	100 (67)	80 (54)		43 (64)	41 (55)	
Female	49 (33)	69 (46)		24 (36)	33 (45)	
TNM stage (%)						
I	34 (23)			17 (25)		
II	54 (36)			25 (37)		
III	35 (23)			15 (22)		
IV	26 (17)			10 (15)		

### Performance of ctDNA-based methylation signatures in training and test sets

**Fig. 2:**
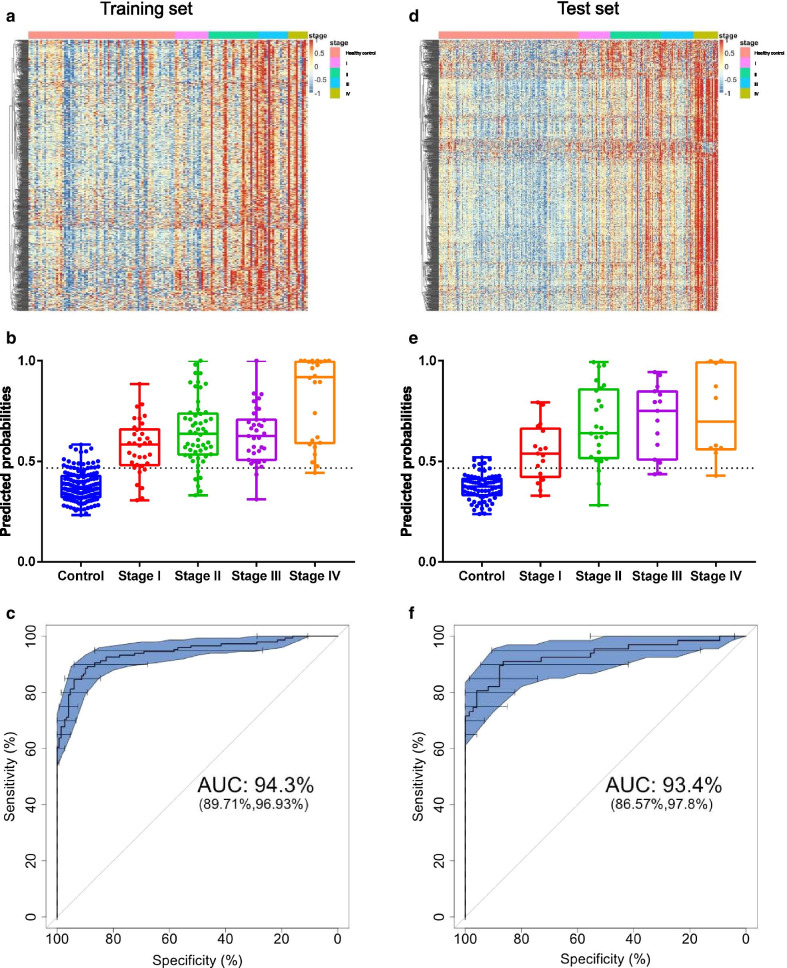
Predicted probabilities of the early detection model in training and test sets. **a** Heatmap depicted the clustering of methylation markers differentially methylated between participants with colorectal cancer and healthy controls in the training set. **b** Predicted probabilities for healthy controls and cancer patients with Stages I–IV in the training set. **c** ROC curve and corresponding AUC for the early detection model in the CRC diagnosis in the training set. **c** Heatmap depicted the clustering of methylation markers differentially methylated between participants with colorectal cancer and healthy controls in the test set. **d** Predicted probabilities for healthy controls and cancer patients with Stage I–IV in the test set. **e** ROC curve and corresponding AUC for the early detection model in the CRC diagnosis in the test set

In the training set, the methylation levels for all selected specific methylation blocks are depicted in Fig. 2a. The predicted probabilities are demonstrated in Fig. 2b. The predicted probabilities were increased with stage and were significantly higher in cancer than healthy controls (*P* < 0.05, Fig. 2b). The methylation signatures demonstrated an area under curve (AUC) of 94.3% (95% CI, 89.7–96.7%) (Fig. 2c). As determined via the Youden’s index method, the specificity was 89.3% (83.1–93.7%) and sensitivity was 88.6% (82.4–93.2%) (Table 2). The sensitivities also increased with stage and were 79.4% (62.1–91.2%), 88.9% (77.3–95.8%), 91.4% (76.9–98.2%) and 96.2% (80.3–99.9%) for patients with stage I, stage II, stage III and stage IV, respectively. The specificity was 94.6% (89.7–97.6%) and sensitivity was 80.6% (73.2–86.6%) when the cutoff value was determined by high specificity, while the specificity was 85.9% (79.3–91.0%) and sensitivity was 89.9% (83.9–94.3%) when the cutoff value was determined by high sensitivity (Table 2).

**Table 2:** Performance of the early detection model based on ctDNA methylation in the training and test sets

Patient group	Youden's index best			High specificity			High sensitivity		
	Tested	Positive	Positive rate (%)	Tested	Positive	Positive rate (%)	Tested	Positive	Positive rate (%)
*Training set*									
Stage I	34	27	79.4% (62.1–91.2%)	34	23	67.6% (49.4–82.6%)	34	28	82.4% (65.5–93.2%)
Stage II	54	48	88.9% (77.3–95.8%)	54	46	85.2% (72.8–93.4%)	54	48	88.9% (77.4–95.8%)
Stage III	35	32	91.4% (76.9–98.2%)	35	28	80.0% (63.1–91.6%)	35	33	94.3% (80.9–99.3%)
Stage IV	26	25	96.2% (80.3–99.9%)	26	23	88.5% (69.7–97.5%)	26	25	96.2% (80.3–99.9%)
All cancer	149	132	88.6% (82.4–93.2%)	149	120	80.6% (73.2–86.6%)	149	134	89.9% (83.9–94.3%)
Healthy control	149	16	10.7% (6.3–16.9%)	149	8	5.4% (2.4–10.3%)	149	21	14.1% (9.0–20.7%)
*Test set*									
Stage I	17	12	70.6% (43.9–89.6%)	17	11	64.7% (38.2–85.8%)	17	12	70.6% (44.0–89.7%)
Stage II	25	22	88.0% (68.6–97.4%)	25	22	88.0% (68.8–97.5%)	25	22	88.0% (68.6–97.4%)
Stage III	15	13	86.7% (59.6–98.4%)	15	12	80.0% (52.0–95.7%)	15	13	86.7% (59.6–98.4%)
Stage IV	10	9	90.0% (55.2–99.7%)	10	9	90.0% (55.4–99.7%)	10	9	90.0% (55.6–99.7%)
All cancer	67	56	83.6% (72.5–91.6%)	67	54	80.6% (69.1–89.3%)	67	56	83.6% (72.4–91.6%)
Healthy control	74	6	8.1% (3.0–16.8%)	74	3	4.1% (0.8–11.4%)	74	6	8.1% (3.0–16.9%)

In the test set, there were also significant differences in methylation signatures between healthy controls and cancers (Fig. 2d). The predicted probabilities of the early detection model are demonstrated in Fig. 2e. Similar phenomenon was observed that the predicted probabilities were also increased with stage (Fig. 2e). The AUC of the early detection model was 93.4% (95% CI, 86.6–97.8%, Fig. 2f). The performance was consistent with that in the training set with the specificity of 91.9% (83.2–97.0%) and the sensitivity of 83.6% (72.5–91.6%) (Table 2) with the cutoff value determined by Youden’s index. The sensitivities also increased with increasing stage: the sensitivities were 70.6% (43.9–89.6%) in stage I, 88.0% (68.6–97.4%) in stage II, 86.7% (59.6–98.4%) in stage III and 90.0% (55.2–99.7%) in stage IV (Table 2).

With the optimal cutoff value determined in the training set, we further tested the model in combined training and test sets. The specificity was 90.1% (85.4–93.7%) and sensitivity was 87.0% (81.8–91.2%) (Additional file 1: Fig. 1). Altogether, these results showed the similar performance of ctDNA-based methylation in the training and test sets.

### Performance of early detection model in unmatched population

To further test the performance of the early detection model in a larger uncontrolled population, we then applied the model in unmatched population together with benign colorectal disease and advanced adenoma. Similar with the above results, the sensitivities were acceptable for all stages with 73.8% (63.1–82.8%) in stage I, 89.2% (82.8–93.8%) in stage II, 85.9% (75.0–93.3%) in stage III and 95.6% (84.8–99.5%) in stage IV (Additional file 1: Fig. 2). The sensitivities of the early detection model in the total CRC patients stratified by age (≤ 55 years old, vs. > 55 and ≤ 65 years old vs. > 65 years old) were similar (Additional file 1: Table 1), suggesting age did not significantly influence the robust of the early detection of colorectal cancer. In terms of benign colorectal disease and advanced adenoma, it was no surprise that the specificity decreased from 92.2% (88.8–94.9%) in healthy controls to 69.2% (56.5–80.1%) in benign colorectal disease and 41.7% (15.3–72.4%) in advanced adenoma. To be noted, advanced adenoma is a precancerous lesion of CRC and thus shares similar methylation signatures with CRC. Advanced adenoma also needs to be surgically removed, and thus, the high positive ratio in advanced adenoma of our early detection model is acceptable. However, the positive ratio was relatively high in benign colorectal disease; thus, we further explored the underlying reason by dividing benign disease according to pathological behavior. The probabilities for each type of benign disease are also demonstrated in Additional file 1: Figure S3. The positive ratios were 33.3% (1/3) for hyperplastic/inflammatory polyps, 33.3% (1/3) for serrated adenoma, 42.9% (6/13) for mixed hyperplastic adenomatous polyps, 9.5% (2/21) for tubular adenoma, 35.3% (6/17) for villous tubular adenoma and 71.4% (5/7) for villous adenoma, respectively. These results suggested that pre-altered mucosa especially villous adenoma might share similar methylation signatures with CRC. These patients with positive results need to be under surveillance to observe whether CRC will occur.

### Comparison between our early detection model and previously published methylation SEPT9

The methylation sites for SEPT9 were also included in the targeted methylation panel we used in the present study, so we further compared our early detection model with the mSEPT9 model. The cutoff value for SEPT9 was also obtained by Youden’s index method in the training set and then validated in the test set. The AUC for SEPT9 was 65.5% (53.5–76.8%) in the training set and 67.3% (58.9–74.6%) in the test set (Additional file 1: Fig. 4A, B), which were worse than the performance of the present early detection model (*P* < 0.001). The specificity was comparable between the present early detection model and SEPT9 (90.1% [85.4–93.7%] vs. 90.6% [86.0–94.1%] in total matched population), while the sensitivities were higher in the present early detection model than SEPT9 (87.0% [81.8–91.2%] vs. 41.2% [34.6–48.1%], *P* < 0.001) (Additional file 1: Fig. 4C), suggesting that the present early detection model may have better utility than mSEPT9 alone in clinic, which needs to be further studied in the future.

## Discussion

Early detection of CRC is a major challenge to improve patients’ survival and widen the window of therapeutic intervention. However, current screening strategies suffer from low compliance rates due to uncomfortable and time-consuming procedures. In the present study, we assessed the ability of ctDNA-based methylation, a noninvasive approach, to early detect CRC. The sensitivity was 88.6% (82.4–93.2%) and 83.6% (72.5–91.6%) in training and test sets (Table 2), respectively. Our results suggested that ctDN-based methylation was a promising approach in early detection of CRC.

Several ctDNA-based methylation markers have been investigated for early detection for CRC, such as SEPT9. The methylation of SEPT9 was proposed for discriminating CRC from healthy control with the AUC of 0.8 [26], and yielding a sensitivity of 48.2% and specificity of 91.5% in another large, prospective trail of 7941 patients [24]. SEPT9 DNA methylation model based on our data showed an inferior performance compared with our early detection model in the present study, with AUC of 0.67 in test set. Another methylation marker cg10673833 yielded the sensitivity of 89.7%, 33.35 and 21.9% in CRC, advanced precancerous lesion, and non-advanced denoma, respectively, in screening of high-risk population [17]. The cd-score, a classifier integrating 9 methylation markers, demonstrated the sensitivity of 87.9% and specificity of 89.6%, for discriminating CRC from healthy controls [17], which is similar to our early detection model.

One of the most important attributes of an early detection model is the ability to detect early-stage cancers. The sensitivity was 27–50% for stage I CRC in the Circulating Cell-free Genome Atlas (CCGA) study [23]. The sensitivity of SEPT9 was 45–47% for stage I CRC [24]. The sensitivities suggested that more than 50% stage I CRC were under-detected and were far from satisfied. Compared with these studies, our early detection model performed better for stage I patients with a sensitivity of 79.4% in the training set and 70.6% in the test set. Altogether these results suggested that our model might have a promising utility in clinic, which requires further studies to confirm.

In benign colorectal disease, the positive ratio was 30.8%. We hypothesized that several precancerous lesions shared similar methylation signatures with CRC; thus, several benign colorectal diseases were detected as positive. When categorized with detailed pathological behavior, the positive ratio was relatively low in tubular adenoma. Tubular adenoma is a common benign colorectal disease with low risk of carcinogenesis. The positive ratios were relatively high in villous tubular adenoma and villous adenoma. Patients with villous adenoma are at high risk of CRC and are recommended to perform colonoscopy or surgical removal [27]. The positive ratios in benign colorectal carcinoma are consistent with the risk of carcinogenesis. For participants with hyperplastic/inflammatory polyps and serrated adenoma with little risk of CRC carcinogenesis, the positive ratios were also relatively high; however, there were only 3 participants with hyperplastic/inflammatory polyps and 3 participants with serrated adenoma. Thus, the present early detection model needs to be further tested in hyperplastic/inflammatory polyps and serrated adenoma with a larger sample size. We are uncertain that these false positive individuals who were not diagnosed as CRC by colonoscopy presently would develop carcinoma in the future; however, classifying them as false positives provides the most conservative approach to interpretation of the data. Further studies on this population are needed. The positive ratio was also high for advanced adenomas. Advanced adenomas are precursor lesions of CRC. These results suggest that early-detection model-based identification and removal of advanced adenomas may be a path to reducing CRC incidence.

In our present study, we matched patients with CRC and healthy controls by age. However, in previous studies, age did not always match between cases and controls because healthy controls are usually younger than cancer patients. Previous study showed that with the increase of age, about 5% of the CpG sites exhibited a significant change of methylation in humans [25, 28], and approximately half of the genes where age-related methylation occurs are the same genes that are involved in the tumorigenesis of CRC [22]; thus, it is important to match CRC cases and healthy controls with age. Without matching, one cannot tell whether the performance of the methylation model is achieved due to the difference of age instead of CRC-specific methylation. In our study, the performance of our early detection model was similar between unmatched and matched cases and controls, one reason may be that only a small fraction (~ 2%) methylation sites change with age [29], so these are not the key point for differentiating the CRC from healthy controls. Another possibility is that these targeted methylation sites analyzed in our study are presupposed based on the differential expression of methylation sites between CRC and paracancerous tissue, engendering ineluctable insufficiency and deviation of marker screening. However, the influence of age in CRC early detection model needs to be further investigated.

Several limitations may impede the interpretation of our results. Firstly, the CRC patients were individuals with known cancers, most of which were diagnosed on the basis of symptoms. The fraction of stage I tumors will probably be higher among asymptomatic, screened individuals, and consequently the sensitivity of detection in a screening population might be less than reported here. Secondly, the healthy controls and cases were from different hospitals, which might introduce bias into this study. Thirdly, the lack of an independent validation cohort requires further validation of the early detection model. Fourthly, we compared the methylation of SEPT9 and the early detection model generated from the same panel instead of running another independent SEPT9 test, which may induce some biases. The last, we only focused on CRC in the present study, while the feature of methylated ctDNA allows us to identify tissue-of-origin. Future studies are needed to study the utility of methylated ctDNA in multiple carcinomas.

## Conclusion

In summary, in this present study from training to test, we built an early detection model of CRC based on ctDNA methylation. Whether or not the early detection model could be used as a screening tool needs to be further studied in a larger population with the risk of CRC. Based on these results, a prospective Pan-CanceR Early DetectIon ProjeCT (PREDICT study) has been registered in ClinicalTrials.gov with NCT number (NCT04383353) and is ongoing.

### Participants and methods

#### Study design and participants

This is a prospective, multicenter, case–control study. Participants with diagnosed CRC, benign colorectal disease or advanced adenoma in the Cancer Centers of Changhai Hospital Affiliated to the Naval Medical University and the Sixth Affiliated Hospital, Sun Yat-sen University Guangdong Gastrointestinal Hospital were enrolled from June 2017 to September 2019. CRC, benign colorectal disease or advanced adenoma was diagnosed by a pathologist according to pathological analysis and colonoscopy examinations. Advanced adenoma was defined as adenoma ≥ 1 cm, with tubulovillous/villous histology or with high-grade dysplasia. CRC, benign colorectal disease or advanced adenoma participants were excluded if the diagnosis was not further confirmed by the pathological results or the patients were previously treated. Healthy controls were defined as participants without the previous history or presence of carcinoma or critical illness including chronic obstructive pulmonary disease, hepatitis, liver cirrhosis and colorectal disease and were recruited from Chongqing University Cancer Hospital from November 2019 to December 2019. All healthy volunteers would receive routine healthy checkup including blood tests, urine tests, blood biochemical tests, electrocardiograms, low-dose CT and abdominal ultrasound. Participants with normal test results would be included in the study. All study participants were between 40 and 75 years old.

We performed a cautious sample selection procedure to keep age balance between case and control samples. For tumor patients, samples with age above 60 took 63.0% which was much more than 17.1% for healthy controls. One hundred and sixteen from 209 patients older than 60 were randomly filtered from modeling. Meanwhile, healthy samples with age below 50 took 43.8% which was much more than 9.2% for case group. One hundred and ten from 146 younger than 50 were randomly filtered from modeling. After matching by age, CRC patients and healthy controls were randomly divided into training and test datasets with a 2:1 ratio to build an early detection model. The performance of the early detection model was further tested in total unmatched population and participants with benign colorectal disease and advanced adenoma.

This study was approved by the Ethics Committee of Changhai Hospital Affiliated to the Naval Medical University, the Sixth Affiliated Hospital (2017-072) and recorded by the Ethics Committee of Sun Yat-sen University Guangdong Gastrointestinal Hospital and Chongqing University Cancer Hospital and adhered to local ethics. All participants provided informed consent.

#### Sample collection, storage and processing

The procedures for sample collection, storage and processing were as previously described [30]. In brief, 8–10 ml of whole blood samples for each participant were collected by Cell-Free DNA Streck tubes and centrifuged at 1600 g for 20 min at room temperatures to obtain the plasma. All plasma was stored at -80℃. The QIAamp Circulating Nucleic Acid Kit (551114, Qiagen, Valencia, CA, USA) was used to extract ctDNA from plasma.

#### Target methylation sequencing and data preprocessing

To enrich the CRC-related methylated variation signal, we selected thousands of specific CpG sites as the target based on the 450 K microarray data of colorectal tumor samples, normal samples and white blood cells from The Cancer Genome Atlas (TCGA) and Gene Expression Omnibus (GEO) datasets. The methylation sites of SEPT9 were also included. A capture-based method was used to cover these CpG sites. We generated bisulfite sequencing library with the brELSATM method (Burning Rock Biotech, Guangzhou, China). The target libraries were quantified by real-time PCR and sequenced on NovaSeq 6000 with 1000× target depth on average.

With the raw sequencing data, several bioinformatics tools including Trimmomatic, BWA-meth and samblaster were applied to the alignment and caller of reads as the downstream analysis. Since differential methylated region consisting of multiple CpG sites played more important roles than a single CPG site in cancer detection as reported in the literature [31], we defined CpG sites with close genomic distance and highly correlation in methylation level as special methylation block. As a result, we generated a matrix of regional methylation value of 8090 methylation blocks for all samples.

### Statistical analysis

We assumed that the methylation early detection model may improve the diagnostic results (area under curve, AUC) from 80 to 90%. With the marginal error of the estimated AUC not exceed to 5% with 95% confidence level, the minimal sample size for both case and control groups was 106. Continuous variables were described with mean ± SD and were compared by two-sided *t* tests or the Mann–Whitney U test. Categorical variables were described with number (percentages) and compared by Chi-square test or Fisher’s exact test. On the detection model building, fivefold cross-validation was applied to the training data to avoid over-fitting and supporting vector machine was selected as a two-category classifier to distinguish cases and controls. The criteria for model selection and parameter optimization depended on maximizing the area under the receiver operating characteristics curve (ROC) that was also used to evaluate the performance of the early detection model. The optimal cutoff value for the early detection model was determined by Youden’s index. Cutoff point with high specificity was determined by max_c_ {Se(c) + 2*Sp(c)−1}, and max_c_ {2*Se(c) + Sp(c)−1} with high sensitivity. The 95% confidence interval for sensitivities and specifies were generated using the exact binomial distribution. Comparisons between ROC curves were performed using the Hanley-McNeil method. *P* = 0.05 was set as the level of significance and all *P* values were two-sided. All statistical analyses were performed using R 3.4.2.

## Supplementary information

**Additional file 1.** The performances of the early detection model in the subgroup analysis including total matched population, unmatched population, colorectal-related benign disease and stratification by age and the comparison between the early detection model and previously reported mSEPT9 model.

## Data Availability

The data used in the present study is available upon request to the corresponding author Wei Zhang (weizhang2000cn@163.com).

## Abbreviations

AUC: Area under curve
CRC: Colorectal carcinoma
ctDNA: Circulating tumor DNA
FIT: Fecal immunochemical testing
FOBT: Fecal occult blood testing
ROC: Receiver operating curve
TCGA: The Cancer Genome Atlas
