# Supplementary material for: Discovery and validation of methylation signatures in blood-based circulating tumor cell-free DNA in early detection of colorectal carcinoma: a case–control study
Source: Clin Epigenetics. 2021 Feb 3;13:26. doi: 10.1186/s13148-020-00985-4 (PMC7856810; doi:10.1186/s13148-020-00985-4)
Supplement: Supplementary file 1 — Additional file 1. The performances of the early detection model in the subgroup analysis including total matched population, unmatched population, colorectal-related benign disease and stratification by age and the comparison between the early detection model and previously reported mSEPT9 model. [file 13148_2020_985_MOESM1_ESM.docx]

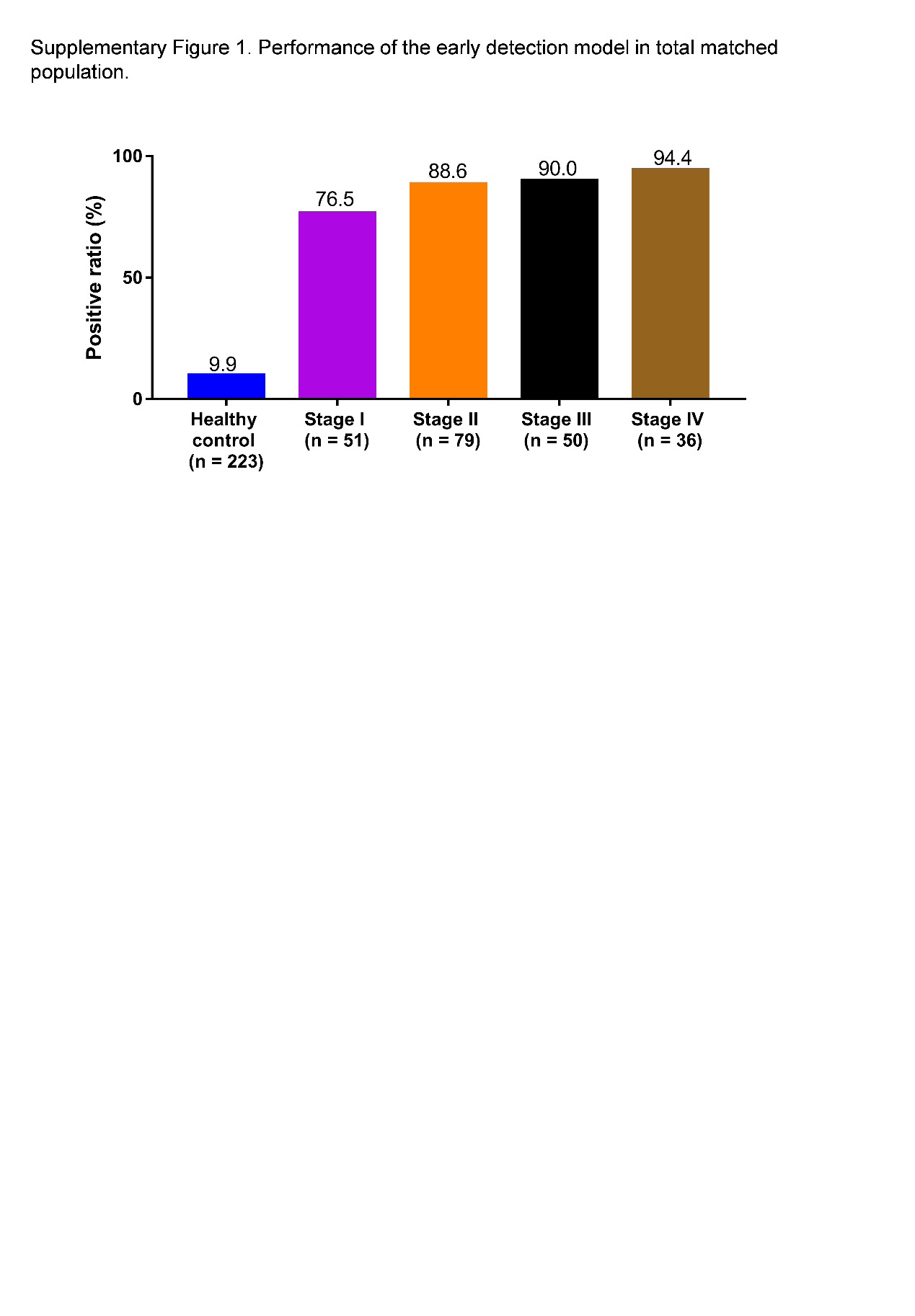


**Supplementary Figure S1. Performance of the early detection model in total matched population.**


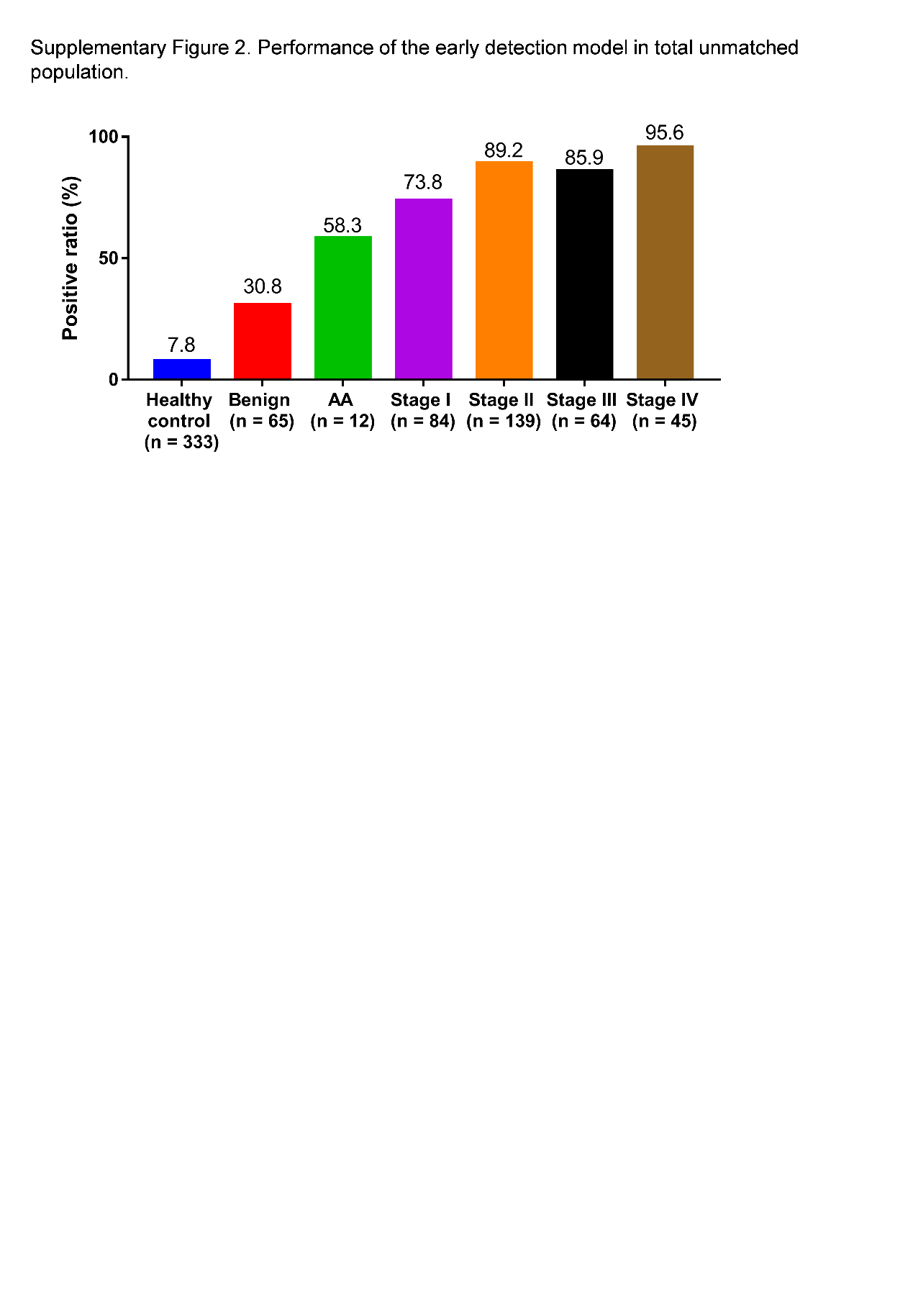


**Supplementary Figure S2. Performance of the early detection model in total unmatched population.**


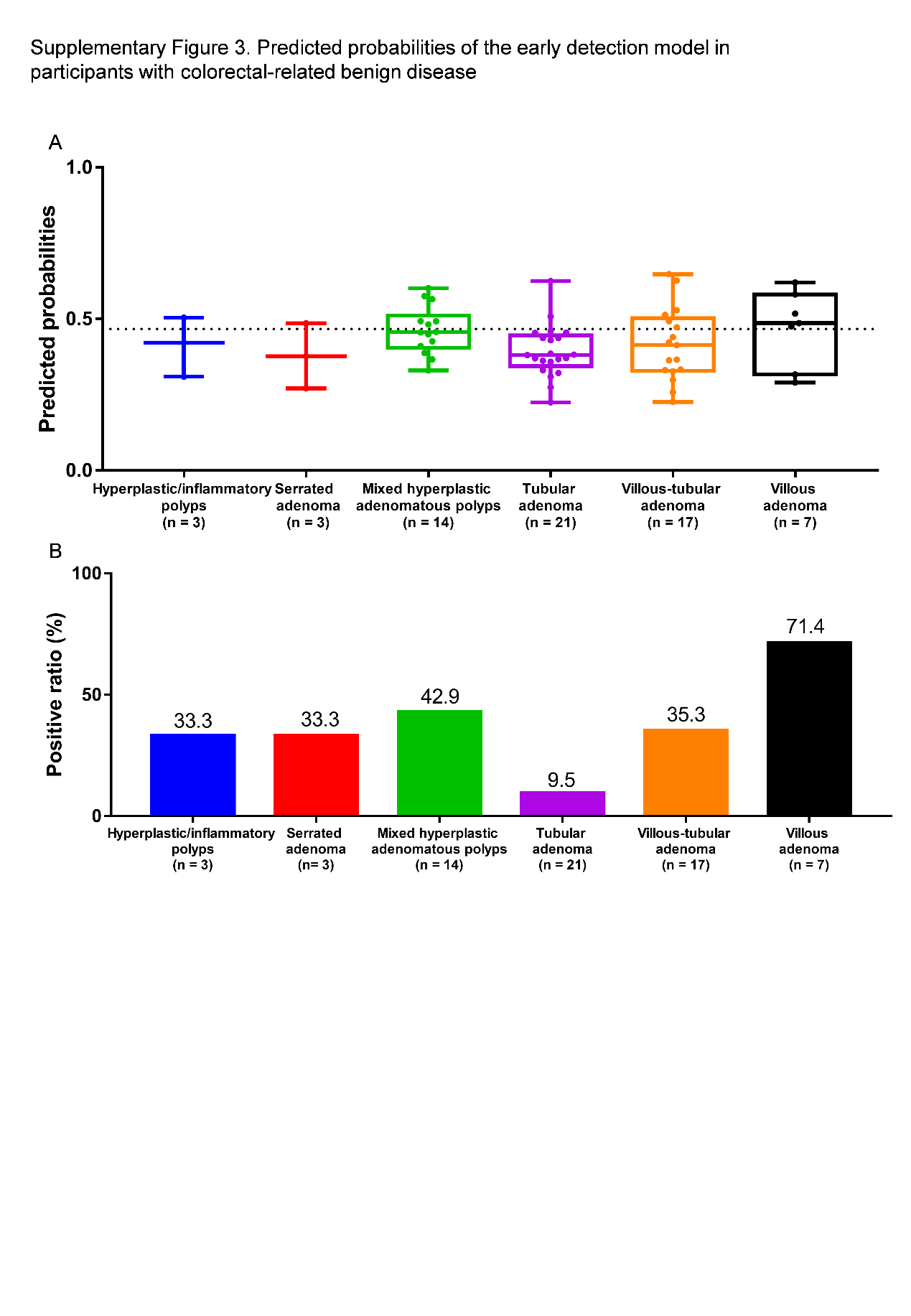


**Supplementary Figure S3. Predicted probabilities of the early detection model in participants with colorectal-related benign disease.** (A-B) Predicted probabilities (A) and positive ratios (B) of the early detection model in participants with colorectal-related benign disease.


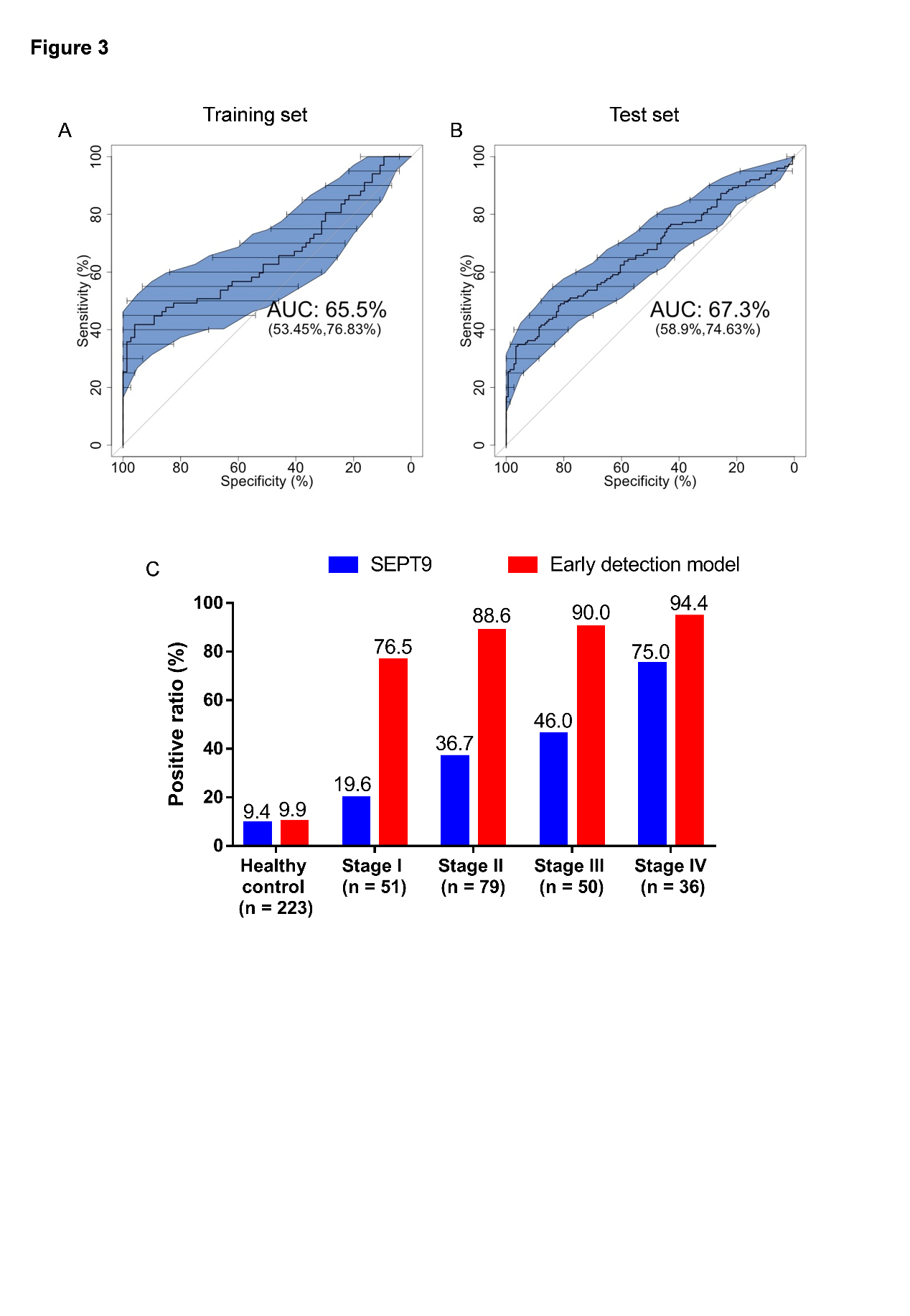


**Supplementary Figure S4. Comparison between the early detection model and previously reported mSEPT9 model.** (A-B) ROC curves and corresponding AUC for mSEPT9 in CRC diagnosis in the training (A) and test (B) sets. (C) Comparison of the performances of the present early detection model and mSEPT9 in early detection of CRC.

**Supplementary Table 1. Sensitivities of the early detection model in the total unmatched CRC patients stratified by age.**

| **Patient Group** | **Age** | | | | | | | | | | |
| --- | --- | --- | --- | --- | --- | --- | --- | --- | --- | --- | --- |
|  | **≤ 55 years old** | | |  | **>55 and ≤65 years old** | | |  | **>65 years old** | | |
|  | **Tested** | **Positive** | **Positive rate (%)** |  | **Tested** | **Positive** | **Positive rate (%)** |  | **Tested** | **Positive** | **Positive rate (%)** |
| I | 20 | 14 | 70.0% (45.6%-88.2%) |  | 29 | 23 | 79.3% (60.2%-91.9%) |  | 35 | 25 | 71.4% (53.7%-85.4%) |
| II | 29 | 24 | 82.8% (64.2%-94.1%) |  | 60 | 53 | 88.3% (77.5%-95.2%) |  | 50 | 47 | 94.0% (83.4%-98.7%) |
| III | 16 | 11 | 68.8% (41.4%-89.0%) |  | 25 | 22 | 88.0% (68.7%-97.4%) |  | 23 | 22 | 95.7% (78.2%-99.9%) |
| IV | 17 | 15 | 88.2% (63.5%-98.5%) |  | 16 | 16 | 100.0% (79.5%-100.0%) |  | 12 | 12 | 100.0% (73.6%-100.0%) |
| All cancer | 82 | 64 | 78.0% (67.5%-86.4%) |  | 130 | 114 | 87.7% (80.7%-92.8%) |  | 120 | 106 | 88.3% (81.2%-93.5%) |
